# Post COVID-19 Pandemic and Intimate Partner Violence: Family Physician to Its Rescue

**DOI:** 10.21315/mjms2023.30.6.16

**Published:** 2023-12-19

**Authors:** Ramasamy Chidambaram, Indumathi Sivakumar

**Affiliations:** Department of Prosthodontics, Faculty of Dentistry, SEGi University, Selangor, Malaysia

**Keywords:** family physician, facial injury, intimate partner abuse, referral

## Abstract

COVID-19 has created panic waves wherein the world is under test and Malaysia is no exception. Intimate partner violence (IPV) is one among the exhaustive list and seen rise in cases especially after the lockdown where families must spend more time with each other. Good news that the movement restrictions are lifted, meanwhile it is likely that family physicians can expect more admissions of IPV. Reports from the Ministry of Health Malaysia state the last year cases have noticed a spike of 42%. Thereby the present communication aims to keep the family physician informed on the rising IPV related injuries. Our prime concern is about the reluctant victims that remain undetected. In reality, situations become tougher especially when victim is accompanied by her perpetrator. Being the first person to contact victim, the family physician should utilise this post-pandemic as a potential strategy to identify the voiceless victims in perpetrators presence.

## Introduction

In the euphoria of modern lifestyles, we often forget the social connection with our family. Among such overlooked problems is the relationship with the intimate partner (IP). When things go sour with the IP, a sacred relationship within four walls can transform into intimate partner violence (IPV) and might even reach the court’s doors. IPV is a grave issue in which the IP engages in physical, sexual, verbal or psychological violence or stalking. The victims of IPV are mostly women. Based on global estimates, the experience of physical IPV accounts for up to 30% of a woman’s lifetime (1, 2). Malaysia is not spared from the rising IPV cases with a prevalence of 4.94%–35.9% (1). Alarmingly, the national prevalence is higher than the global average (1). Lower educational background and substance abuse among the perpetrators are the main contributing factors to IPV (1). Apart from the increasing number of IPV annually, the severity of each case is also worsening. However, the conviction rate of IPV remains low, mainly due to under-reporting as many victims perceive this as a sensitive issue and thus, the perpetrators continue to roam free (1, 3). Thus, the main concern lies with many of the reluctant IPV victims who remain undetected. The enhancement of diagnostic measures can potentially reduce the prevalence of IPV. In this matter, family physicians (FPs) play a pivotal role. While the general public may counter that the improvement of knowledge and awareness of IPV among healthcare providers is sufficient, the situations become tougher in actual practice, especially when the victim is accompanied by the perpetrator. Furthermore, because of the massive lockdown during the COVID-19 pandemic, IPV cases have been rising because families need to spend more time together under the same roof (4, 5). This can be observed from the trend of domestic complaints in Malaysia, whereby it rose by 57% from 250 in February 2020 to 5,388 between March 2020 and September 2020 based on the report by the Women’s Aid Organisation (WAO) (6) and Talian Kasih (National Crisis Hotline) Malaysia (7). While it is indeed good news that movement restrictions have been eased, it is projected that FPs will receive more complaints and cases of IPV. According to the Ministry of Health Malaysia Report, there was a spike in IPV cases by 42% in 2022 (8). Therefore, FPs must be made aware of rising IPV-related injuries and identify potential strategies to assist these voiceless victims, especially in the presence of the perpetrators.

### The Family Physician’s Profession

From the professional perspective, the rule of thumb is not to ask any questions on IPV in the presence of a perpetrator as it may create an additional threat to the patient. International literature suggests that the region of the head, neck, and face is most susceptible to injury during IPV (9–11). Thus, it is vital to take note of any visible facial injuries, including swollen face, finger-print marks, bruises on the cheek and ear, bite marks, black eye and zygomaticomaxillary complex fractures that may raise suspicion during routine physical examination (12, 13). The wounds could have probably occurred during cooking, managing sharp utensils or from accidental fall. Nevertheless, until the patient discloses any abuse, the injuries should be considered suspicious. At the same time, any injuries inside of on the neck and face, below the ear lobe and on top of the shoulders should also raise suspicion of inflicted trauma. While the perpetrator’s presence may create an unfavourable atmosphere, the right strategy is to wait for the right opportunity. An ideal way to divert the perpetrator’s attention is by requesting him to complete the registration process at the reception. The FP can use this precious time to inquire whether these facial injuries are accidental or intentional. Thus, it is imperative for other medical team members to be aware of potential IPV cases.

However, each patient is different. A few would respond but the majority would continue to suffer in silence. Interpersonal communication between a suspected victim and the physician is necessary to alleviate the associated fears because a short grace time and a hesitant victim may not lead to the desired results. Earlier studies have discussed the limitations from the perspective of both patients and healthcare providers (1, 3). Further explanation of barriers would be beyond the scope of this article as our focus is on the recognition of IPV victims in the perpetrator’s presence.

In the situation whereby the perpetrator disobeys the advice to complete the documentation and insists on staying throughout the entire course of consultation is not ideal. In this situation, the accompanying partner may start talking on the patient’s behalf or even turn aggressive in front of the physician, both are signs that are highly indicative that the accompanying partner is the abuser. Honestly, not everyone’s underlying nature will guide him or her to suspect possible IPV, especially if the focus is solely on the patient’s chief complaint. Thus, there is a higher chance of neglecting the etiology of injuries. As a result, signs such as being overprotective or judgmental, insulting the patient and denying her feelings/wishes are red flags that should trigger the FPs to correlate them with clinical features to ascertain if the patient has been abused.

### The Family Physician’s Resource

In the primary care setting, the best assistance that can be rendered to the victim is to send clear signals despite the presence of the abuser. For example, pictures or slogans indicating support available at the clinic for IPV victims can be displayed on the wall or computer monitors, medical records can be endorsed with the official seal of the clinic highlighting the slogans or giving souvenirs with the intended message when kids are around.

What if victims fail to notice these signals? Another strategy is providing a referral card consisting of all the government or private agencies and non-profit organisations that can be of assistance to IPV victims (Table 1). The services should be included along with the phone numbers, website links, and email IDs, and whether they are available 24/7 or otherwise so that the victims can contact the helpline in an emergency. These organisations are also in contact with the medical team to facilitate any inquiries on the injuries or potential legal matters in the future. However, the referral card must be given in a way without raising the suspicion of the abuser. Placing these cards at the reception is an excellent choice as patients spend more time there. Even if the victims do not take any of the cards due to the fear of being seen by the abuser, they might still be aware of the available channels that can help them. Referral cards in different languages are also useful in view of the multiethnic population in Malaysia. If the victim notices any of the above signals, there is a higher possibility of favourable results. In addition, awareness campaigns among the communities can also indirectly benefit more victims. With the advancement of digital technology, it has become more convenient to spread awareness of IPV among the general public.

### The Family Physician’s Judgement

Having said that, if hypothetically the victim still fails to notice all these tactics or intentionally disregard them, what are the other option available? Truthfully, it depends on the FP’s call and also the existing physical and mental conditions of the victim. They need to tap into their professional experience and practical knowledge to decide whether to raise a concern about this sensitive issue. The entire scenario revolves around the fact even if the perpetrator is unaware that the victim is seeking help, it would still be putting the victim’s or kids’ lives at risk if confidentiality is not maintained. Worse still, physical IPV can sometimes lead to a more severe situation. The 2020 global report shows that 58% of the victims were killed by none other than their IPs (14). What are we still missing here? Time constraints and limited experience could be potential barriers for FPs to perform general screening. As a result, FPs should actively seek more resources and equip themselves with professional IPV training. Validated questionnaires like the Physician Readiness to Manage Intimate Partner Violence Survey (PREMIS) can boost their confidence and prepare them for future cases of IPV (15). More ideally, a Malay version of the questionnaire can provide additional avenues for IPV victims to express their hidden pain. Some studies have shown that writing, especially expressive writing reduces the pain of IPV victims (16) by providing them with a self-healing platform to break their silence.

### Future Family Physicians 2050

By 2030, mental illness is projected to be the greatest public health concern, with domestic violence being one of the common causes (7). On a separate note, more than 90% of the world’s population will have internet access by 2050 (17). With that, doctors can better keep track of their patients by means of online technology and maintain regular follow-ups with IPV victims. Remote monitoring can help assure that the victims receive immediate attention and care when the need arises. To familiarise themselves with the new norm, FPs should attend short courses to update their knowledge on medical and legal aids available for IPV victims (18) as well as to provide echo training for fellow medical officers.

## Conclusion

Last but not least, as healthcare personnel, FPs play a vital role in the mitigation of IPV given that they are often the first point of contact with the victims. Apart from a strong foundation in basic clinical sciences, FPs must also cultivate concerns for the patient’s mental health. Continuous medical education with clear objectives and benefits should be emphasised to attract the attention of busy practitioners so that they can be motivated to be involved in assisting IPV victims. Another effective approach is via the incorporation of technology, for example, the online platform of MyGovernment is one of the best online platforms for all services concerned with life (19). Nevertheless, despite the robust features and state-of-the-art technology applications, it is important to remember that every healthcare worker should remain reactive in fostering mutual trust with their patients. After all, medical education is not only about acquiring knowledge, it is also about the need to be empathetic to help patients to restore the relationship in their complex lives.

## Figures and Tables

**Table 1:** Referral card

Referral card
Women’s Aid Organisation WAO Hotline at +603 7956 3488 SMS/WhatsApp at TINA +6018 988 8058 General enquiries at +603 7957 5636/0636 E-mail: info@wao.org.my Website: https://wao.org.my/
All Women’s Action Society AWAS Helpline at +603 78770224 (9 am to 5.30 pm) General enquiries at +603 78774221 (9 am to 5.30 pm) E-mail: telenita@awam.org.my Website: https://www.awam.org.my/
Social Welfare Department Tel: +603 2697 1090 Fax: +603 2694 1248 Website: www.jkm.gov.my
Sisters in Islam (non-profit organisation) Tel: +603 7960 3357 (9 am to 5 pm) Tel: +603 22739913/14 (9 am to 5 pm) E-mail: sistersinisslam@pd.jaring.my Website: http://www.sistersinislam.org.my/
Befrienders (non-profit organisation) KL: 03-7956 8145 (24 hours) Ipoh: 05-547 7933 (4 pm to 11 pm) Penang: 04-281 5161 (3 pm to midnight) E-mail: sam@befrienders.org.my Website: https://www.befrienders.org.my/
